# The Use of Elastic Bands in Velocity-Based Training Allows Greater Acute External Training Stimulus and Lower Perceived Effort Compared to Weight Plates

**DOI:** 10.3390/ijerph192416616

**Published:** 2022-12-10

**Authors:** Carlos Babiloni-Lopez, Javier Gene-Morales, Angel Saez-Berlanga, Rodrigo Ramirez-Campillo, Juan Antonio Moreno-Murcia, Juan C. Colado

**Affiliations:** 1 Research Group in Prevention and Health in Exercise and Sport, University of Valencia, 46010 Valencia, Spain; 2 Exercise and Rehabilitation Sciences Institute, School of Physical Therapy, Faculty of Rehabilitation Sciences, Universidad Andres Bello, 7591538 Santiago, Chile; 3 Department of Sport Sciences-Sport Research Centre, Miguel Hernández University, 03202 Elche, Spain

**Keywords:** human physical conditioning, resistance training, muscle strength, musculoskeletal and neural physiological phenomena, exercise, physical fitness

## Abstract

The objective was to compare the mean propulsive velocity (MPV), maximum power (PMAX), heart rate, and rate of perceived exertion (RPE) during the parallel squat using elastic bands (EB) or weight plates (WP) to load the bar. The effect of relative strength on the dependent variables was analysed. Additionally, the potential of the RPE to predict external load parameters was assessed. Eighteen trained volunteers squatted at 40%, 55%, 70%, and 85% of their one-repetition maximum with EB and WP (a total of eight sets) in random order. Dependent variables were measured at the first and last repetition (i.e., 10% velocity loss). Two identical sessions were conducted to assess the reliability of measurements. Compared to WP, EB allowed a significantly greater number of repetitions, MPV, and PMAX, and significantly lower RPE. The RPE of the first repetition was a significant predictor of the external load of the set. The RPE showed good repeatability and was not influenced by the relative strength of athletes. In conclusion, compared to WP, the use of EB allows for greater external load with reduced internal load responses in a wide spectrum of load-based intensities. The potential implications of these novel findings are discussed in the manuscript.

## 1. Introduction

Both weight plates (WP) and elastic bands (EB) have been used in resistance training to gain muscle mass and strength in a wide range of populations such as trained athletes and older adults [1,2,3]. Indeed, both WP and EB may induce similar strength gains (upper limbs: Standardized Mean Difference [SMD] = −0.11; 95% CI = −0.40, 0.19; *p* = 0.48; lower limbs: SMD = 0.09; 95% CI = −0.18, 0.35; *p* = 0.52) [4]. Additionally, resistance training using EB can produce superior training-induced improvements in fatigue resistance, greater increases in serum testosterone and cortisol concentration [5], similar muscle electromyographic activity [6,7], and permit more repetitions [8] compared to resistance training with WP. However, a comparison between using EB or WP regarding maximum power (PMAX) and mean propulsive velocity (MPV) is lacking. Considering the relevance of PMAX and MPV during constant resistance velocity-based training (VBT) [9], more research on this topic is warranted.

Elastic bands are durable, affordable, and easy-to-use resistance-training devices [10,11,12]. As opposed to WP, the elongation coefficient of EB increases the resistance as the bands are elongated (i.e., greater load as the EB tension increase) [13].These different characteristics of EB and WP make it difficult to equate training variables such as repetitions, load, and velocity between both materials. To compensate for these differences between EB and WP, autoregulation strategies could be used [14]. For instance, the rate of perceived exertion (RPE) has been established as a valid and reliable tool for measuring resistance training intensity using WP [15] and EB [16]. In this regard, the RPE has a high accuracy (i.e., 86%) for estimating the relative load (percentage of one-repetition maximum [%1RM]) in parallel squats with WP [17]. Bearing in mind that the relative load or kilograms used are difficult to measure with EB, the question arises as to whether RPE could be a feasible tool for trainers to estimate different training programming variables (e.g., kilograms, %1RM, repetitions, MPV) in resistance exercises using EB to load the bar. To the best of our knowledge, no previous research has evaluated the potential of the RPE to estimate the relative load in squats performed with EB.

Furthermore, a recent meta-regression [18] showed that sociodemographic and training-related parameters such as age, sex, and training status had no significant effects on the RPE. However, as far as we are aware studies analysing whether the relative strength ratio (i.e., kilograms of 1RM divided by body weight) could influence the RPE values in resistance training with EB are scarce.

Therefore, the purpose of the present study was to compare the difference in external (number of repetitions, MPV, and PMAX) and internal (RPE and heart rate) load variables between using EB or WP to load the bar in parallel squats in a wide range of intensities (40%, 55%, 70% and 85% 1RM) and considering the relative strength of participants. As a secondary objective, the RPE of the first repetition was evaluated as a potential predictor of the external load of each set.

Bearing in mind that EB provide less weight in the deepest phases of the squat, it was hypothesized that the use of EB would allow for (i) greater external load values and (ii) lower internal load values, with the participants’ relative strength not influencing the internal load parameters. Additionally, we hypothesized that (iii) the RPE would be a significant predictor of the external load parameters analysed.

## 2. Materials and Methods

### 2.1. Study Design

This study is part of a large research project designed to analyse the effects of different methods of applying EB to load the bar (i.e., at the standing position or above the sticking point) in the parallel squat performed at maximum velocity and the validation of the RPE of the first repetition to quantify the load of the set. This research was carried out in the Sports Performance Laboratory of the Faculty of Physical Activity and Sports Sciences of the University of Valencia (Spain).

The present study employed a quasi-experimental design. Each participant provided informed consent and was free to withdraw from the study at any time. All procedures were applied in accordance with the requirements listed in the 1975 Declaration of Helsinki and its amendment in 2013, and all experimental protocols were conducted according to country laws and approved by the Ethics Committee of the University of Valencia (Spain) for peer review (H20190325095509). Two 90 min familiarization sessions were completed the first week, and two experimental sessions for assessment of the dependent variables the following week. A 1:3 participant-researcher ratio was used in all sessions. All measurements were taken by the same researchers, at the same time of day, and were always conducted in the same laboratory.

### 2.2. Procedures

The familiarization sessions were employed to conduct the anthropometric measurements, evaluate the one-repetition maximum (1RM) load, and familiarize the participants with the RPE scales and the procedures. In the first session, anthropometric measurements (height, weight, and % body fat) were performed with a stadiometer (IP0955, Invicta Plastics Limited, Leicester, UK) and a bioelectrical impedance scale (Tanita model BF-350, Tokyo, Japan). Additionally, each participant’s squat range of movement was measured with a goniometer to identify the height at which the bands had to be attached to the bar and the squat depth. A standardized 10 min warm-up including joint mobility, bodyweight exercises, jogging, and dynamic stretching was conducted. At this point, the 1RM (see Section 2.2.3. 1RM Calculation) was indirectly calculated. Thereafter, four sets (at 40%, 55%, 70%, and 85% 1RM; a total of eight sets) using WP to load the bar and four using EB were performed in random order (i.e., sealed envelopes). The second familiarization session consisted of performing the same eight sets in a different order with the aim of the participants gaining more experience with the RPE scales and the study procedures.

After 72 h, the two experimental sessions were carried out separated by 48 h. In both experimental sessions, a total of eight sets using WP or EB to load the bar with loads between 40–85% 1RM were performed in random order. Homogeneous rests of five minutes were allowed between sets or conditions. Meanwhile, MPV and PMAX were recorded with an Encoder (see Section 2.2.5. Movement Velocity Measurement and Maximum Power) for the first and last repetition (10% velocity loss). RPE was verbally mentioned by the participants after the first and last repetition at the standing position. Additionally, heart rate, kilograms at the standing position, and repetitions were immediately recorded when each set was finished. Participants were instructed to not look at the Smith machine before performing each condition to avoid biases. They rested in an adjacent space separated from the Smith machine by a partition screen while listening to music with headphones to avoid getting audible information about the next condition. According to recent literature [19,20], participants were allowed to choose the music they wanted to listen to. Constant feedback was given, and two trained spotters were standing on both sides of the bar to ensure proper execution and to encourage maximum effort.

#### 2.2.1. Participants

The sample size was determined using G* Power 3.1 software [21]. The calculation indicated a sample size of 18 volunteers to meet a statistical power of 0.80, α = 0.05, a correlation coefficient of 0.50, nonsphericity correction of 1, and an effect size of 0.60. This a priori analysis was performed to reduce the probability of type II error by determining the minimum number of participants required to reject the null hypothesis at the *p* < 0.05 level of confidence [22].

Therefore, 18 physically active, healthy participants (12 men and 6 women; age: 23.7 ± 3.4 years; body mass index: 23.3 ± 2.6 kg/m^2^; body fat percentage: 12.4 ± 3.8%; 1RM: 104.8 ± 27.7 kg; relative strength: 1.5 ± 0.2) volunteered to participate. Only participants with a minimum of six months of resistance training experience and familiarized with the squat exercise were accepted as eligible to participate. Participants who had suffered any type of muscle, ligament, or tendon injury one year prior to the study were excluded. Participants were instructed not to consume any type of stimulating food or drink three hours before each session, not to exercise at a high intensity involving the lower limbs ≥24 h before each test, and to sleep ≥8 h.

#### 2.2.2. Execution Protocol and Exercise Description

**Figure 1:**
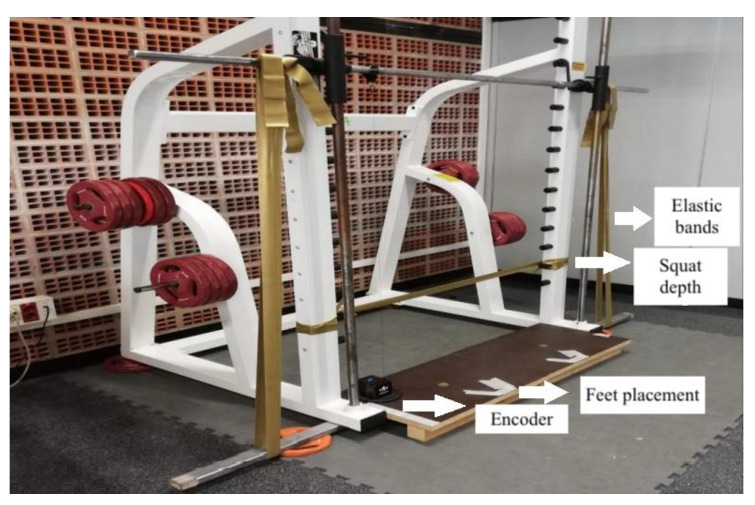
Measurement setting. Smith machine used to perform parallel squats with elastic bands or weight plates. In this case, it is shown its use with elastic bands.

A Smith machine (Powerline, PSM14X, Body-Solid, Chicago, IL, USA) was used to perform parallel squats [23]. Elastic bands (CLX bands, TheraBand^®^; Hygenic Corporation, Akron, OH, USA) or weight plates (ranging from 1.25 to 20 kg; Olive, Manresa, Spain) were used to load the barbell. A digital scale (Salter, Model 9179 SV3R, Barcelona, Spain) with a precision of 50 g and an adjustable strut with a range of 95–170 cm (Piher, Model 30011, Logroño, Spain) was used for the measurement of the weight provided by the elastic resistance. The height of the standing position of each participant was marked to measure the kilograms provided by the EB at this height (Figure 1). The bar was placed on the upper platform of the strut at this height, while the lower platform of the strut was placed in the centre of the scale to obtain the proportional load provided by the EB (the bar weighed 20 kg). A digital metronome (Metronome Beats, London, UK) set at 60 beats/min paced the repetition rate to ensure that all the sets were always performed at a standardized pace. The feet-position was set at the shoulder width of each participant. A tape was used to mark the same feet-position for every set. Joint angles were set using a manual goniometer (Baseline^®^, New York, NY, USA) to establish the depth of the squat (a parallel line between the trochanter and lateral epicondyle of the femur and the floor, i.e., 81.12 ± 3.74 knee joint angle degrees). At this position, a horizontal elastic band was attached to the Smith machine for each participant to execute properly the exercise. Participants were asked to perform an eccentric phase of two seconds until contacting (midthigh) the horizontal elastic band (deepest squat position) and the concentric phase as fast as possible. After the concentric phase, participants were instructed to maintain the position for two seconds (2 beats) to indicate the RPE of the first repetition. The set was finished when the encoder indicated a 10% loss of intra-set speed. At this point, participants reported the RPE again.

#### 2.2.3. 1RM Calculation

Four sets with WP were performed to indirectly calculate the weight for 1RM. The four sets were as follows: (i) 20 repetitions without additional weight (the bar weighed 20 kg); (ii) 15 out of 20 possible repetitions; (iii) 10 out of 13 possible repetitions; (iv) a load that would allow between 8–12 repetitions to calculate the 1RM. O’Connor or Epley [24,25] formulas were used when <10 repetitions or when 10 or more repetitions were performed, respectively. If a participant performed a number of repetitions that fell outside of the aforementioned range (8–12 repetitions), another set with an altered load was performed. A rest of 5 min was allowed between the sets. More time was permitted depending on the perception of the participants [26].

#### 2.2.4. Heart Rate Measurement

Heart rate monitors (Polar FT1, Polar Electro, Tampere, Finland) were attached to the chest of each participant. The heart rate was collected immediately after the completion of the last repetition of every set.

#### 2.2.5. Movement Velocity Measurement and Maximum Power

A linear position and velocity transducer (Speed4Lift, Madrid, Spain; measurement frequency at 1000 Hz) with high reliability and validity (coefficient of variation [CV] = 2.61%; intraclass correlation coefficient [ICC] = 0.87) [27] was used to measure the MPV, PMAX, and the total number of repetitions in each set. The weight offered by the EB at the standing position was used to obtain PMAX values in the conditions with EB.

#### 2.2.6. Perceived Exertion Values Measurement

RPE for the overall body was measured with the OMNI-RES for weight training [15] and the OMNI-Resistance Exercise Scale of Perceived Exertion for EB [16]. A detailed explanation of both scales was provided to the participants, where 0 was considered ‘no effort’ and 10 ‘maximum effort’. Participants had to answer the following question: How hard do you feel your muscles are working? Both scales were in clear view to the participants during the entire session.

#### 2.2.7. Relative Strength

The relative strength ratio was measured by dividing the 1RM load (in kilograms) by the body weight (in kilograms). The relative strength classification used in this study was based on previous research [28]. Specifically, none of the participants of this study presented poor relative strength levels (<1.0), seven participants presented normal relative strength levels (between 1.0 and 1.5), and 11 participants presented high relative strength levels (>1.5).

### 2.3. Statistical Analyses

Statistical analyses were performed using commercial software (SPSS, version 26.0, SPSS Inc., Chicago, IL, USA). All variables were checked for normality using the Shapiro–Wilk test. Almost all the variables presented a normal-Gaussian distribution. Only the number of repetitions performed in three conditions, and the RPE showed a nonnormal distribution. In this regard, a parametric analysis of variance (ANOVA) was used due to its robustness [29]. Results are reported as mean and standard deviation (SD). The level of significance was set at *p* < 0.05.

A three-way mixed ANOVA of repeated measures was used to assess the influence of each intensity (40%, 55%, 70%, and 85% 1RM), the material used to load the bar (WP and EB), and relative strength level (medium [1.0–1.5] and high [>1.5]) on the heart rate and the number of repetitions performed. A four-way mixed analysis of variance (ANOVA) of repeated measures was carried out to determine the influence of each intensity (40–85% 1RM), repetition (first repetition and last repetition), material used to load the bar (WP and EB), and relative strength level (medium [1.0–1.5] and high [>1.5]) on the MPV, PMAX, and RPE. For the effect size analysis, partial eta squared values (η_p_^2^) were obtained derived from ANOVA and were interpreted as low (<0.04), moderate (0.04–0.13) and large (>0.13). Planned pairwise comparisons were conducted using the Bonferroni correction to evaluate differences. The effect size of the post hoc comparisons was calculated by means of Cohen’s d, which was interpreted as a low (<0.50), moderate (0.50–0.79), or large effect (≥0.80) [30].

Additionally, a categorical linear regression analysis was carried out with the RPE of the first repetition as a predictor for the MPV, load, and number of repetitions.

Finally, the reliability of load, heart rate, number of repetitions, MPV, PMAX, and RPE measures were assessed through the intraclass correlation coefficient (ICC). As previously suggested, ICC values were interpreted as poor (<0.50), moderate (0.50–0.75), good (0.75–0.90) and excellent (>0.90) reliability, based on the lower bound 95% confidence interval [31].

## 3. Results

### 3.1. Number of Repetitions and Heart Rate Values

Table 1 shows the values for the number of repetitions and heart rate. The results of the ANOVA showed that the material used to load the bar influenced the number of repetitions (F(_1,16_) = 35.03, *p* < 0.001, η_p_^2^ = 0.69) and heart rate (F(_1,16_) = 17.89, *p* < 0.001, η_p_^2^ = 0.53). The interaction between intensity*material used to load the bar only showed a significant effect on the heart rate (F(_3,48_) = 2.97, *p* < 0.05, η_p_^2^ = 0.16). There were no significant effects of the relative strength or other interactions on the heart rate and the number of repetitions (*p* > 0.05).

### 3.2. Mean Propulsive Velocity, Maximum Power, and Rating of Perceived Exertion

Table 2 shows the MPV, PMAX, and RPE at the first repetition and last repetition (i.e., 10% velocity loss). The ANOVA showed that the repetition analysed (first or last repetition) influenced the MPV (F(_1,15_) = 11.26, *p* < 0.001, η_p_^2^ = 0.43), PMAX (F(_1,15_) = 4.42, *p* < 0.05, η_p_^2^ = 0.23), and RPE (F(_1,15_) = 5.84, *p* < 0.05, η_p_^2^ = 0.28). The interaction intensity*material used showed a significant effect on MPV (F(_3,45_) = 32.48, *p* < 0.001, η_p_^2^ = 0.68) and PMAX (F(_1.82,27.30_) = 51.71, *p* < 0.001, η_p_^2^ = 0.77), but not in RPE (*p* = 0.09). The interaction intensity*repetition*material used only showed a significant effect on the RPE (MPV: *p* = 0.61; PMAX: *p* = 0.13; RPE: F(_3,45_) = 4.40, *p* < 0.001, η_p_^2^ = 0.23). The interactions intensity*relative strength (MPV: F(_1.78,24.90_) = 10.43, *p* < 0.001, η_p_^2^ = 0.43; PMAX: *p* = 0.48; RPE: *p* = 0.57), material*relative strength (MPV: F(_1,14_) = 36.20, *p* < 0.001; η_p_^2^ = 0.72; PMAX: F(_1,14_) = 48.68, *p* < 0.001, η_p_^2^ = 0.78; RPE: *p* = 0.11), and repetition*relative strength (MPV: F(_1,14_) = 4.68, *p* < 0.05, η_p_^2^ = 0.25; PMAX: F(_1,14_) = 22.44; *p* < 0.001, η_p_^2^ = 0.62; RPE: *p* = 0.16) showed a similar trend with a non-significant effect on the RPE. The pairwise post hoc comparisons from the interaction intensity*relative strength showed significantly higher MPV at 40% and 55% 1RM (*p* < 0.05) and significantly greater PMAX at 40% to 85% 1RM (*p* < 0.05) for the participants with high relative strength levels. However, there were no significant between-group differences in RPE (*p* < 0.05).

### 3.3. Prediction of Load, Number of Repetitions and MPV through the RPE

Categorical linear regression analysis was used to predict the number of repetitions, load, and MPV in the first and last repetition. The results are shown in Table 3. Specifically, there was a very significant (*p* < 0.001) linear association between RPE (independent variable) and the number of repetitions, load, and MPV (dependent variable). Table 4 shows score equivalences in dependent variables from categorical linear regression analysis. Load, the number of repetitions, and MPV (first and last repetition) values were determined from RPE values (from 1 to 10 points).

### 3.4. Intersession Reliability for Load, Repetitions, Heart Rate, MPV, PMAX, and RPE

The internal consistency analysis for load, repetitions, heart rate, and first and last repetition MPV, PMAX, and RPE across different sessions showed a good (ICC = 0.75–0.90) to excellent (ICC > 0.90) reliability. The intersession reliability was similar for EB and WP in all the study variables. However, the ICC of the number of repetitions and the first and last repetition RPE were lower when WP were used compared to EB (repetitions ICC: 0.66 [WP] vs. 0.79 [EB]; first repetition RPE ICC: 0.66 [WP] vs. 0.89 [EB]; last repetition RPE ICC: 0.72 [WP] vs. 0.87 [EB]).

## 4. Discussion

The aims of this study were (1) to compare differences in variables of the external and internal load in parallel squat performed with WP or EB attached to the bar; (2) to assess the influence of the relative strength in the study variables; (3) to evaluate the RPE as a potential predictor of the external load. The main finding was that external load variables (i.e., MPV, PMAX, and repetitions) were higher in EB conditions at each intensity while internal load responses (i.e., heart rate and RPE) were lower when EB were used to load the bar compared to WP conditions. Considering that the differences in heart rate were significant only in two conditions, these results are partially in line with our first hypothesis. Regarding the second aim, the relative strength ratio did not influence the internal load variables, which confirms our second hypothesis. Finally, according to our third hypothesis, Table 3 and Table 4 present significant regression equations to predict parameters of the external load through the RPE of the first repetition.

Bearing the aforementioned results in mind, it is worth discussing the outputs of this research in light of other empirical evidence that addressed the dependent variables of the present study. Therefore, this discussion is organized presenting first the differences between using EB or WP to load the bar in parallel squats regarding the external and internal load. Second, the effects of the relative strength on the study variables. Finally, the last section discusses the potential use of the RPE of the first repetition to quantify the external load in squats.

### 4.1. External Load: Differences between Using Elastic Bands or Weight Plates to Load the Bar

The use of EB to load the bar in parallel squats allowed the participants to perform more repetitions than the use of WP did (average repetitions of the four intensities: 10.73 [EB] vs. 8.41 [WP]). As presented in Table 1, significant differences (*p* < 0.05) between using EB or WP to load the bar appeared at 55% 1RM (d = 0.88), 70% 1RM (d = 1.77), and 85% 1RM (d = 1.89). This could be due to the elongation coefficient of EB [7]. Since EB provide fewer kilograms in the lower parts of the squat and more in the upper part [32], EB favour the overcoming of the sticking region with fewer kilograms [8], allowing a greater number of repetitions when the bar is loaded with EB instead of WP. Additionally, this could be due to the muscular and resistance profile of the quadriceps and parallel squats, respectively, which favours the implementation of EB for improving the performance in parallel squats in terms of repetitions and muscular activation [33,34].

As reported in previous articles [35,36], the use of EB can be a good option to increase parameters such as muscle activity, PMAX, velocity, and the ratio of force development. Accordingly, in our study, using EB to load the bar allowed participants to perform at significantly higher velocities (*p* < 0.05; see Table 2) in all intensities compared to the use of WP (40% 1RM: d = 0.98; 55% 1RM: d = 2.61; 70% 1RM: d = 2.43; and 85% 1RM: d = 3.82). As happened with the number of repetitions, these greater values for the EB could be due to the weight reduction throughout the sticking region, in which the upward velocity of the barbell decreases [7,8]. Therefore, the athletes could be moving the barbell at a greater velocity during the initial degrees of the concentric phases when EB were used, and the mean velocity of the lift would be greater compared to the use of WP to load the bar.

The power-load relationship in the parallel squat has traditionally been described by a parabolic shape [37]. Specifically, the maximal power production in squats is achieved with moderate loads (i.e., from >30% to <70% of 1RM) [38]. Our results (see Table 2) show a power peak at 70% 1RM and a diminishment at 85% 1RM with the constant load, which is in accordance with Soriano et al. [38]. However, the parabolic shape does not exist when EB are used to load the bar because of a constant increase of power from 40% to 85% 1RM. Regarding the comparison between the use of EB and WP, squatting with EB allowed participants to produce significantly greater power (*p* < 0.05) compared to the use of WP (40% 1RM: d = 0.45; 55% 1RM: d = 0.83; 70% 1RM: d = 1.09; 85% 1RM: d = 1.51). While caution should be applied until more scientific evidence arrives, this could mean that squatting with EB is a useful tool for improving PMAX in athletes. Although PMAX was measured at the standing position of the parallel squat in both conditions and the load was equated at this position, a plausible explanation for the higher values in the EB condition could be the variable resistance that EB offer. According to this, the load provided by the EB during the lower phases of the squat was inferior to that provided by the WP. Therefore, PMAX throughout these lower phases of the squat may have been greater in the conditions that used WP to load the bar compared to the use of EB [35]. Further studies to explore the power in 100% 1RM and calculate the exact load at each part of the squat range of movement in variable resistance conditions are still warranted.

### 4.2. Internal Load: Differences between Using Elastic Bands or Weight Plates to Load the Bar

Regarding the heart rate (see Table 1), it was significantly (*p* < 0.05) higher when WP were used compared to using EB at 40% 1RM (d = 0.68) and 55% 1RM (d = 0.77). Although being the same exercise and doing more repetitions in the EB squat, the participants had to lift a constant load during the whole range of movement in the WP squat. Therefore, fatigue was higher in WP conditions, which has a greater impact on heart rate [39].

Previous research has shown very strong associations between the RPE and %1RM (r = 0.88 to 0.91) and strong inverse relationships between MPV and RPE (r = −0.79 to −0.87) [40]. However, a gap in this field exists when variable resistance training devices (i.e., EB) are used. In our study, the RPE values for EB conditions were always lower compared to WP conditions at each intensity (40% 1RM: d = 1.71; 55% 1RM: d = 1.31; 70% 1RM: d = 1.23; 85% 1RM: d = 1.13). This might be due to the weight reduction in the lower phases of the squat when EB were used.

### 4.3. The Use of the Rate of Perceived Exertion of the First Repetition to Quantify the External Load in Squats

Finally, the linear regression showed significant values (r) above 0.49 in all the variables (see Table 3). Similar results were obtained in the prediction of the number of repetitions and %1RM through the RPE in both conditions WP and EB. The values shown in Table 4 can serve as the first guide for sports scientists and coaches to develop their field training with an easy-to-use tool (RPE), which has been validated for both WP and EB resistance training.

As demonstrated by previous expert research [41,42], the relative strength ratio has a significant effect on the outcomes of athletes in objective variables such as MPV and PMAX. Accordingly, in our study, the participants with high relative strength levels obtained higher MPV and PMAX in some conditions. However, the relative strength did not significantly affect the subjective variable RPE (i.e., non-significant differences between conditions). Previous investigations had already shown no significant effects of gender, age, and training status on RPE values [43,44,45]. In addition to that, our study shows that RPE could also be used regardless of people’s fitness level (i.e., relative strength). Furthermore, the potential use of RPE to quantify the external load in EB resistance training is reinforced by the positive intersession reliability values obtained (ICC for the RPE of the first repetition = 0.89).

### 4.4. Limitations

Although all the procedures were carefully designed and conducted, there are several limitations to mention. First, the standing position of each participant was used to equate the load in WP and EB parallel squats. However, the use of EB varies the load during the remaining range of movement. Formulas, such as that used by the encoder to calculate PMAX, are affected by the different weight that EB provide in each part of the range of movement. In this regard, a better agreement between the scientific community is necessary to establish different formulas in encoders to adapt them to the EB idiosyncrasy. While caution should be applied until more scientific evidence arrives, the results presented in Table 4 serve as a reference for sports scientists and strength and conditioning coaches who want to have a guide to program external load training parameters with EB.

## 5. Conclusions

To the best of our knowledge, this is the first study to compare parameters of velocity-based training when EB or WP are used to load the bar in squats. In conclusion, compared to WP, the use of EB to load the bar in parallel squats allows training sessions with a greater number of repetitions, greater power, and velocity, lower RPE, and heart rate values in a wide spectrum of intensities commonly used in real-life training programs. Finally, this study highlights the usefulness of the RPE of the first repetition to quantify the external load (i.e., number of repetitions, %1RM, and MPV) of the set. With these data (see Table 4), trainers or athletes could maintain or modify the kilograms added to the bar with EB depending on the RPE value reported by the athlete in the first repetition, with no need to weigh the bar.

## Figures and Tables

**Table 1:** Number of repetitions and heart rate in each condition.

Intensities	Number of Repetitions WP †	Number of Repetitions EB †	Heart Rate WP (bpm)	Heart Rate EB (bpm)
40% 1RM	15.22 (2.07)	16.22 (3.57)	165.65 (16.14)	155.18 (14.64) *
55% 1RM	9.89 (3.36)	12.33 (2.03) *	164.82 (12.69)	154.76 (13.34) *
70% 1RM	5.44 (1.42)	8.39 (1.88) *	157.59 (13.68)	153.76 (13.47)
85% 1RM	3.11 (1.02)	6.00 (1.91) *	153.82 (12.51)	150.12 (11.94)

Data are expressed as mean (standard deviation). * Significant differences (*p* < 0.05) compared to WP. † Significant differences (*p* < 0.05) between each intensity. 1RM: one-repetition maximum; EB: elastic bands; WP: weight plates; bpm: beats per minute.

**Table 2:** MPV, P_MAX_, and RPE values for the first and last repetition.

		40% 1RM	55% 1RM	70% 1RM	85% 1RM
**MPV (m/s)**	First Repetition WP	0.84 (0.08) ^#^	0.70 (0.06) †^#^	0.58 (0.07) †^#^	0.45 (0.07) †^#^
	First Repetition EB	0.94 (0.13)	0.90 (0.09)	0.80 (0.10) †	0.73 (0.09) †
	Last repetition WP	0.72 (0.08) ^#^	0.60 (0.05) †^#^	0.49 (0.07) †^#^	0.38 (0.06) †^#^
	Last repetition EB	0.82 (0.12)	0.78 (0.09)	0.69 (0.10) †	0.63 (0.08) †
**P_MAX_ (W)**	First Repetition WP	349.26 (115.92) ^#^	404.41 (125.58) †^#^	423.87 (128.09) †^#^	385.83 (112.55) ^#^
	First Repetition EB	408.83 (150.95)	530.22 (184.01) †	591.49 (187.40) †	649.60 (219.79) †
	Last repetition WP	300.17 (101.67) ^#^	343.39 (102.99) †^#^	354.42 (106.70) †^#^	340.98 (98.11) ^#^
	Last repetition EB	355.34 (132.63)	461.06 (161.20) †	514.73 (168.61) †	568.31 (189.90) †
**RPE**	First Repetition WP	2.72 (1.71) ^#^	3.94 (1.29) ^#^	6.11 (1.81) †^#^	7.61 (1.65) †^#^
	First Repetition EB	1.28 (0.75)	2.56 (1.15) †	4.11 (1.41) †	5.72 (1.90) †
	Last repetition WP	6.06 (1.21) ^#^	6.22 (1.35) ^#^	7.67 (1.53) ^#^	8.44 (1.42) †^#^
	Last repetition EB	3.22 (1.26)	4.28 (1.27) †	5.78 (1.59) †	6.67 (1.53) †

Data are expressed as mean (standard deviation). † Very significant differences (*p* < 0.001) compared with 40% 1RM; ^#^: Significant differences compared to elastic bands. MPV: mean propulsive velocity; PMAX: maximum power; 1RM: one-repetition maximum; RPE: rating of perceived exertion; EB: elastic bands; WP: weight plates.

**Table 3:** Prediction of load, number of repetitions, %1RM, and MPV values from the first repetition rating of perceived exertion score (linear regression).

Variable	Condition		r	R^2^ (SEE)	Significance (*p*-Value)	Regression Equation
Load (kg)	WP		0.59	0.35 (20.97)	<0.001	35.21 + (6.07 × RPE)
	EB		0.49	0.24 (22.62)	<0.001	46.01 + (5.90 × RPE)
Number of repetitions	WP		0.67	0.45 (3.80)	<0.001	15.46 + (−1.38 × RPE)
	EB		0.69	0.48 (3.35)	<0.001	15.78 + (−1.48 × RPE)
%1RM	WP		0.76	0.58 (11.07)	<0.001	36.31 + (5.14 × RPE)
	EB		0.78	0.61 (10.63)	<0.001	41.53 + (6.14 × RPE)
MPV (m/s)	WP	First repetition	0.75	0.57 (0.11)	<0.001	0.89 + (−0.05 × RPE)
		Last repetition	0.58	0.34 (0.12)	<0.001	0.90 + (−0.05 × RPE)
	EB	First repetition	0.68	0.46 (0.10)	<0.001	0.99 + (−0.04 × RPE)
		Last repetition	0.54	0.29 (0.10)	<0.001	0.90 + (−0.03 × RPE)

SEE: standard error of estimation. %1RM: percentage of one-repetition maximum; MPV: mean propulsive velocity; EB: elastic bands; WP: weight plates.

**Table 4:** Rating of perceived exertion (RPE) equivalences for load, number of repetitions, %1RM, and MPV from the categorical linear regression analysis.

RPE	1	2	3	4	5	6	7	8	9	10
Load EB (kg)	51.91	57.81	63.71	69.61	75.51	81.41	87.31	93.21	99.11	105.01
Load WP (kg)	41.28	47.35	53.42	59.49	65.56	71.63	77.70	83.77	89.84	95.91
Number of repetitions EB	14.30	12.82	11.34	9.86	8.38	6.90	5.42	3.94	2.46	0.98
Number of repetitions WP	14.08	12.70	11.32	9.94	8.56	7.18	5.80	4.42	3.04	1.66
%1RM EB	47.67	53.81	59.95	66.09	72.23	78.37	84.51	90.65	96.79	102.93
%1RM WP	41.45	46.59	51.73	56.87	62.01	67.15	72.29	77.43	82.57	87.71
MPV first repetition EB	0.95	0.91	0.87	0.83	0.79	0.75	0.71	0.67	0.63	0.59
MPV first repetition WP	0.84	0.79	0.74	0.69	0.64	0.59	0.54	0.49	0.44	0.39
MPV last repetition EB	0.87	0.84	0.81	0.78	0.75	0.72	0.69	0.66	0.63	0.60
MPV last repetition WP	0.85	0.80	0.75	0.70	0.65	0.60	0.55	0.50	0.45	0.40

Rating of perceived exertion data are expressed for each score in the OMNI-RES EB scale. kg: kilograms; %1RM: percentage of one-repetition maximum; MPV: mean propulsive velocity; EB: elastic bands; WP: weight plates.

## Data Availability

All data generated or analysed during this study are included in this published article (Table 1, Table 2, Table 3 and Table 4). The databases are available upon reasonable request to the corresponding author.
